# Differentiable Processing of Objects, Associations, and Scenes within the Hippocampus

**DOI:** 10.1523/JNEUROSCI.0263-18.2018

**Published:** 2018-09-19

**Authors:** Marshall A. Dalton, Peter Zeidman, Cornelia McCormick, Eleanor A. Maguire

**Affiliations:** Wellcome Centre for Human Neuroimaging, Institute of Neurology, University College London, London WC1N 3AR, United Kingdom

**Keywords:** associations, fMRI, hippocampus, objects, scenes, space

## Abstract

The hippocampus is known to be important for a range of cognitive functions, including episodic memory, spatial navigation, and thinking about the future. However, researchers have found it difficult to agree on the exact nature of this brain structure's contribution to cognition. Some theories emphasize the role of the hippocampus in associative processes. Another theory proposes that scene construction is its primary role. To directly compare these accounts of hippocampal function in human males and females, we devised a novel mental imagery paradigm where different tasks were closely matched for associative processing and mental construction, but either did or did not evoke scene representations, and we combined this with high-resolution functional MRI. The results were striking in showing that different parts of the hippocampus, along with distinct cortical regions, were recruited for scene construction or nonscene-evoking associative processing. The contrasting patterns of neural engagement could not be accounted for by differences in eye movements, mnemonic processing, or the phenomenology of mental imagery. These results inform conceptual debates in the field by showing that the hippocampus does not seem to favor one type of process over another; it is not a story of exclusivity. Rather, there may be different circuits within the hippocampus, each associated with different cortical inputs, which become engaged depending on the nature of the stimuli and the task at hand. Overall, our findings emphasize the importance of considering the hippocampus as a heterogeneous structure, and that a focus on characterizing how specific portions of the hippocampus interact with other brain regions may promote a better understanding of its role in cognition.

**SIGNIFICANCE STATEMENT** The hippocampus is known to be important for a range of cognitive functions, including episodic memory, spatial navigation, and thinking about the future. However, researchers have found it difficult to agree on the exact nature of this brain structure's contribution to cognition. Here we used a novel mental imagery paradigm and high-resolution functional MRI to compare accounts of hippocampal function that emphasize associative processes with a theory that proposes scene construction as a primary role. The results were striking in showing that different parts of the hippocampus, along with distinct cortical regions, were recruited for scene construction or nonscene-evoking associative processing. We conclude that a greater emphasis on characterizing how specific portions of the hippocampus interact with other brain regions may promote a better understanding of its role in cognition.

## Introduction

There is long-standing agreement that the hippocampus is essential for supporting memory, especially long-term episodic or autobiographical memory (Scoville and Milner, 1957; Squire, 1992; Clark and Maguire, 2016) and for facilitating spatial navigation (O'Keefe and Nadel, 1978). More recently, the hippocampus has been linked with other roles, including scene perception (Graham et al., 2010), short-term memory (Hartley et al., 2007; Hannula and Ranganath, 2008), constructing mental representations of scenes (Maguire and Mullally, 2013; Zeidman and Maguire, 2016), imagining the future (Hassabis et al., 2007; Schacter et al., 2012), decision-making (Mullally and Maguire, 2014; McCormick et al., 2016), and mind-wandering (Karapanagiotidis et al., 2017; McCormick et al., 2018). In addition, accumulating evidence suggests that different hippocampal subfields are preferentially recruited during specific cognitive processes (Eldridge et al., 2005; Zeidman et al., 2015b; Guzman et al., 2016; Berron et al., 2016; Hodgetts et al., 2017; Dimsdale-Zucker et al., 2018).

Numerous theories, including the relational theory and scene-construction theory, attempt to describe how the hippocampus may support such a seemingly diverse range of functions. The relational theory proposes that the hippocampus is required for the binding of arbitrary relations among individual elements within an experience or associating items within a context regardless of whether these associations are couched within a spatial framework (Cohen and Eichenbaum, 1993; Konkel and Cohen, 2009). This view has much in common with other theories that place associative processing at the heart of hippocampal function, namely, the binding of item and context model (Ranganath, 2010), the domain dichotomy model (Mayes et al., 2007), the configural theory (Rudy and Sutherland, 1995), the constructive episodic simulation hypothesis (Roberts et al., 2018), and the high-resolution binding hypothesis (Yonelinas, 2013).

In contrast, the scene construction theory posits that a prime function of the hippocampus is to construct internal models of the world in the form of spatially coherent scenes. Summerfield et al. (2010) and Mullally and Maguire (2013) found that three objects and a three-dimensional (3D) space are sufficient to form the subjective experience of a scene during mental imagery. This is the operational definition of a scene that we use here. Recently, scene construction has been linked with a specific part of the hippocampus—the anterior medial portion that encompasses the presubiculum and parasubiculum (pre/parasubiculum; Maass et al., 2014; Zeidman et al., 2015a,b; Hodgetts et al., 2016; Zeidman and Maguire, 2016; Dalton and Maguire, 2017).

Our goal in the current study was to directly compare the relational/associative and scene construction theories. We devised a novel mental imagery task in which participants engaged in mental construction of objects, nonscene arrays [three objects and a two-dimensional (2D) space], and scenes (three objects and a 3D space). These tasks were matched for associative processing but, importantly, only the latter evoked the mental experience of a scene representation. This paradigm, therefore, made it possible to examine whether hippocampal recruitment was modulated by the associative processing required for both array and scene construction, or whether the hippocampus was preferentially engaged by scenes. Findings either way would provide novel evidence to inform conceptual debates in the field.

Given previous findings linking the anterior medial hippocampus with scene processing, we predicted that this area would be activated by our scene construction task. We also evaluated the recent relevant prediction, based on anatomical considerations, that the objects task might preferentially activate prosubiculum/CA1 due to direct links with the perirhinal cortex (PRC; Insausti and Muñoz, 2001; Dalton and Maguire, 2017). More widely, we predicted that the retrosplenial cortex (RSC), the posterior cingulate cortex (PCC), and posterior parahippocampal cortex (PHC) would be particularly active during the scene construction task, given their known links with scene processing (Epstein, 2008), while the object and array construction tasks would engage the PRC, given its acknowledged role in object processing (Buckley and Gaffan, 1998; Olarte-Sánchezet al., 2015; Nelson et al., 2016).

## Materials and Methods

### 

#### Participants

Thirty healthy, right-handed participants took part in the study (20 females; mean age, 24 years; SD, 4.12). All had normal or corrected-to-normal vision and gave written informed consent in accordance with guidelines of the University College London research ethics committee.

#### Tasks and stimuli

**Figure 1.**
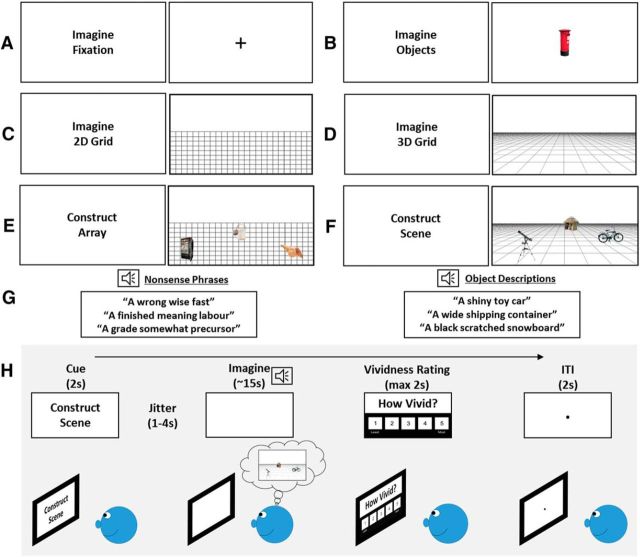
Experimental design. ***A–F***, Representations of how participants engaged in mental imagery during each task, with the text cues on the left of each panel. Note that participants looked at a blank white screen during the imagination phases. The images depicted on the right of each panel are based on what participants said they imagined during the task. ***G***, Examples of object descriptions and nonsense phrases. ***H***, Example of the time line of a single trial.

The functional MRI (fMRI) experiment comprised six separate mental-construction tasks: Imagine Fixation, Imagine Objects, Imagine 2D Grid, Imagine 3D Grid, Construct Array, and Construct Scene (Fig. 1*A–F*). For each task, participants engaged in mental construction with their eyes open while looking at a blank white screen.

For the Imagine Fixation task, participants were asked to imagine a black “plus” sign in the center of a blank white screen (Fig. 1*A*). While imagining the plus sign, participants were auditorily presented with three nonsense phrases (Fig. 1*G*, left), which compromised nonimageable abstract words, spoken one at a time. These were included to mirror the auditory input in the object tasks (see below) while precluding mental imagery. Participants were instructed to try not to interpret the nonsense phrases in any way. This Imagine Fixation task was essentially a rest condition providing participants with a break from the more challenging imagination tasks described below.

For the Imagine Objects task, participants were auditorily presented with three object descriptions (Fig. 1*G*, right) one after another and instructed, when hearing the first object description, to imagine the object alone in the center of the blank white screen (Fig. 1*B*). When hearing the second object description, they were asked to imagine the second object in place of the first in the center of the screen and, when hearing the third object description, to imagine it in place of the second object. During prescan training, participants were instructed and trained to imagine each object in complete isolation.

For the Imagine 2D Grid task, participants were asked to create a mental image of a regular, flat 2D grid covering approximately the bottom two-thirds of the blank screen (Fig. 1*C*). For the Imagine 3D Grid task, participants were asked to create a mental image of a 3D grid covering approximately the bottom two-thirds of the blank screen (Fig. 1*D*). While imagining the grids, participants were auditorily presented with three nonsense phrases, spoken one at a time. The important difference between these tasks is that the 3D grid induces a sense of depth and 3D space.

For the Construct Array task, participants were instructed to first imagine the 2D grid on the bottom two-thirds of the screen. While doing this, participants were auditorily presented with three object descriptions one after another, which they imagined on the 2D grid. More specifically, participants were asked, when hearing the first object description, to imagine the object in an arbitrary location on the 2D grid. When hearing the second object description, participants were asked to imagine it on another arbitrarily chosen location on the 2D grid while maintaining the image of the first object in its original location. When hearing the third object description, participants were asked to imagine it on another part of the 2D grid while maintaining the image of the first two objects in their original positions. We explicitly told participants that the final product of their mental imagery was to be three objects in random locations on a flat 2D grid (Fig. 1*E*).

For the Construct Scene task, participants were instructed to first imagine a 3D grid on the bottom two-thirds of the screen. While doing this, they were auditorily presented with three object descriptions one at a time, which they were asked to imagine on the 3D grid. Specifically, participants were asked, when hearing the first object description, to imagine the object in any location on the 3D grid. When hearing the second object description, participants were asked to imagine it on another location on the 3D grid while maintaining the image of the first object in its original position. When hearing the third object description, participants were asked to imagine it on another part of the 3D grid while maintaining the image of the first two objects in their original locations. The final product of their mental imagery was to be three objects in a simple 3D scene (Fig. 1*F*).

For tasks that required object imagery (Imagine Objects, Construct Array, and Construct Scene), we emphasized the importance of engaging imagination rather than memory for each object. We asked participants to imagine a unique version of each object based on the descriptions alone and, as far as possible, not to recall specific objects that they were familiar with, any personal memories involving the objects, or any places that they might associate with the described objects. We also asked participants not to imagine any movement, even if objects had movable parts, but to create static images of each object in their mind's eye.

For the Imagine 2D Grid and Imagine 3D Grid tasks, participants were instructed to keep their “viewpoint” of the grid fixed and static and not to imagine either the grid moving or themselves moving along the grid. In contrast to the 2D grid, mental imagery of the 3D grid induces a sense of depth and participants were additionally asked not to imagine moving “into” the 3D space in any way.

For the Construct Array and Construct Scene tasks, participants were asked that for each trial, they keep the objects separate from each other so that no objects physically touched and no objects interacted. We asked participants not to add any additional elements but to create the arrays and scenes using only the objects provided. Participants were asked to use the full extent of the grids to place the objects and to continue imagining the objects on the grids for the duration of the imagination period. Also, having imagined all three objects on the grid, participants were asked not to mentally “rearrange” the objects. Rather, they were asked to leave them where they were initially placed in their mind's eye. We asked participants to keep their viewpoint fixed and central and not to imagine themselves or any objects moving in any way. For the Construct Array task, we emphasized the importance of not linking the objects together into a scene but to arbitrarily place the objects in random locations.

Separate audio files were recorded for each object description and nonsense phrase. These were recorded in a sound-proof room and spoken by a male voice in an even tone. Before the experiment, a separate group of participants (*n* = 10) rated each object description on whether it was space-defining or space-ambiguous (Mullally and Maguire, 2011, 2013) and also provided ratings of object permanence (Mullally and Maguire, 2011; Auger et al., 2012). Object descriptions and nonsense phrases were further rated for imageability. The auditory stimuli for each task were all three words in length and carefully matched on a range of specific features.

In relation to the object descriptions, the Imagine Objects, Construct Array, and Construct Scene tasks were matched according to the number of space-defining and space-ambiguous objects (*F*_(2,215)_ = 0.128, *p* = 0.880), ratings of object permanence (*F*_(2,215)_ = 0.106, *p* = 0.899), syllable number (*F*_(2,215)_ = 0.234, *p* = 0.792), and utterance length (*F*_(2,215)_ = 0.014, *p* = 0.986). In addition, the order of presentation of space-defining/space-ambiguous items was balanced across all trials. Object triplets were arranged so that objects within each triplet had no obvious semantic or contextual associations.

In relation to nonsense phrases, syllable number (*F*_(2,215)_ = 1.953, *p* = 0.144) and utterance length (*F*_(2,215)_ = 0.591, *p* = 0.555) were matched across the Imagine Fixation, Imagine 2D Grid, and Imagine 3D Grid tasks. In addition, syllable number (*F*_(5,431)_ = 0.925, *p* = 0.464) and utterance length (*F*_(5,431)_ = 0.658, *p* = 0.656) were matched across all tasks, and the nonsense phrases were rated as significantly less imageable than the object descriptions (*t*_(1,49)_ = 81.261, *p* < 0.001).

In summary, the two tasks of primary interest were the Construct Array and Construct Scene tasks. As described above, these tasks asked participants to listen to three object descriptions and imagine those objects on an imagined 2D or 3D space. With all else being equal in the stimuli, this simple manipulation of space gave rise to mental representations of nonscene arrays (objects and 2D space) and scenes (objects and 3D space). We also included tasks that examined the representation of three objects without a spatial context, where the objects were imagined one after another in the same location on the center of the screen, and the representation of either 2D or 3D space alone without objects. Overall, this novel paradigm allowed us to separately examine the neural correlates of constructing mental representations of objects alone (with no spatial context), of two types of space (2D and 3D space alone with no objects), and of two different combinations of objects and space where only one gave rise to scene representations. Importantly, no visual stimuli were presented during the imagination phase of any task (Fig. 1*H*). Therefore, between-task differences in neural recruitment could not be influenced by differences in visual input.

#### Prescan training

Participants were trained before scanning to ensure task compliance. After listening to the instructions, participants engaged in four practice trials of each task while sitting at a desktop computer in a darkened room. They rated the vividness of mental imagery for each trial on a scale of 1 (not vivid at all) to 5 (extremely vivid). If they gave a rating of ≤3 on any practice trial, they repeated the practice session. When participants rated ≥4 on all practice trials and indicated that they could vividly engage in the mental imagery relevant to each task, they were transferred to the scanner for the experiment.

#### fMRI task

Each trial of the experiment (Fig. 1*H*) comprised a visual cue (2 s), which informed of the task, followed by jitter (1–4 s), and then the imagination phase (∼15 s). During the imagination phase, participants engaged in the mental imagery pertinent to each task while hearing three auditory phrases (either objects or nonsense, depending on the task; Fig. 1*G*) delivered via headphones compatible with MRI scanners (Sensimetrics, model S14). The length of each auditory phrase was ∼2 s followed by a 1 s gap between the presentation of each phrase. After hearing the third auditory phrase, participants had ∼7 s to finalize and maintain the mental image they had created. They were required to do this with their eyes open while looking at a blank white screen. They then rated the vividness of their mental image on a scale of 1 (not vivid at all) to 5 (extremely vivid; maximum, 2 s). Finally, an intertrial interval of 2 s preceded the cue for the next trial. Twenty-four trials were presented for each condition (144 trials in total) in a pseudorandomized order across four separate blocks. Each block lasted ∼15 min and blocks were separated by a brief rest period. It was emphasized to participants that the main objective of the experiment was to create vivid mental images in their imagination. However, to ensure participants were attending throughout, we included an additional 12 catch trials (two per task) across the experiment where participants had to press a button if, within a nonsense or object triplet, they heard a repeated phrase.

#### In-scan eye tracking

As a further measure of task compliance, we used in-scan eye tracking to ensure participants were performing each task according to the instructions. For the Imagine Fixation and Imagine Objects tasks, participants were asked to focus their eyes on the center of the screen where they imagined the plus sign or objects to be. When imagining the 2D and 3D grids, they were asked to move their eyes around where they imagined the grids to be on the screen. For the Construct Array and Construct Scene tasks, participants were required to imagine each of three objects against the imagined 2D or 3D grid respectively. Eye-tracking data were acquired using an Eyelink 1000 Plus (SR Research) eye tracker. The right eye was used for calibration, recording, and analyses. During the imagination phase, the *x* and *y* coordinates of all fixations were recorded. Visualization of fixation locations was performed with Eyelink Data Viewer (SR Research). Eye tracking data from eight participants were unavailable due to technical issues, leaving 22 datasets in the eye tracking analyses.

#### Postscan surprise memory tests

After completing the experiment and leaving the scanner, participants were taken to a testing room and given surprise item and associative yes/no recognition memory tests. Participants first performed an item memory test, where they were auditorily presented with all 216 object descriptions heard during the Imagine Object, Construct Array, and Construct Scene tasks (72 objects per task) and an additional 72 lure items that were not heard during the experiment. Object descriptions were randomized across tasks and were presented one at a time. For each object description, participants were asked to respond “yes” if they thought they heard the object description during the scanning experiment and “no” if they thought they did not.

Participants then performed a more difficult associative memory test. For this, participants were auditorily presented with 72 sets of object triplets (24 sets from each of the Imagine Object, Construct Array, and Construct Scene tasks). Forty-eight of these object triplets (16 from each of the three tasks) had been presented together during the experiment (intact triplets). Twenty-four of the object triplets (eight from each of the three tasks) contained object descriptions presented during the experiment but not together in a triplet (recombined triplets). For each object triplet, participants were asked to respond “yes” if they thought they had heard descriptions of the objects together during the fMRI experiment and “no” if they thought they had not. For both memory tasks, participants also gave a confidence rating on a 1–5 scale for each decision. Also, timing was self-paced (≤5 s each) for both the choices and confidence ratings. Note that we do not include the data from the confidence ratings from the associative memory test as they were, perhaps unsurprisingly, dominated by “guessing” ratings. Memory test data from seven participants were unavailable due to technical issues.

#### Postscan debriefing

Following the memory tests, participants were probed on how they approached each task, and performed a number of ratings as described in the Results and in Table 3.

#### Data acquisition and preprocessing

Structural MRI and fMRI data were acquired using a 3T Siemens Trio scanner (Siemens) with a 32-channel head coil within a partial volume centered on the temporal lobe and including the entire extent of the temporal lobe and all of our other regions of interest. Structural images were collected using a single-slab 3D T2-weighted turbo spin-echo sequence with variable flip angles (Mugler et al., 2000) in combination with parallel imaging to simultaneously achieve a high image resolution of ∼500 μm, high sampling efficiency, and short scan time while maintaining a sufficient signal-to-noise ratio (SNR). After excitation of a single axial slab, the image was read out with the following parameters: resolution, 0.52 × 0.52 × 0.5 mm; matrix, 384 × 328; partitions, 104; partition thickness, 0.5 mm; partition oversampling, 15.4%; field of view, 200 × 171 mm; TE = 353 ms; TR = 3200 ms; generalized autocalibrating partial parallel acquisition (GRAPPA) factor in phase-encoding direction, 2; bandwidth, 434 Hz/pixel; echo spacing, 4.98 ms; turbo factor in PE direction, 177; echo train duration, 881; averages, 1.9. For reduction of signal bias due to, for example, spatial variation in coil sensitivity profiles, the images were normalized using a prescan, and a weak intensity filter was applied as implemented by the scanner's manufacturer. To improve the SNR of the anatomical image, three scans for each participant were acquired, coregistered, and averaged. Additionally, a whole-brain 3D fast low-angle shot (FLASH) structural scan was acquired with a resolution of 1 × 1 × 1 mm.

Functional data were acquired using a 3D echo planar imaging (EPI) sequence, which has been demonstrated to yield improved BOLD sensitivity compared with 2D EPI acquisitions (Lutti et al., 2013). Image resolution was 1.5 mm isotropic and the field of view was 192 mm in-plane. Forty slices were acquired with 20% oversampling to avoid wrap-around artifacts due to imperfect slab excitation profile. The TE was 37.30 ms and the TR was 3.65 s. Parallel imaging with GRAPPA image reconstruction (Griswold et al., 2002) acceleration factor 2 along the phase-encoding direction was used to minimize image distortions and yield optimal BOLD sensitivity. The dummy volumes necessary to reach steady state and the GRAPPA reconstruction kernel were acquired before the acquisition of the image data as described in Lutti et al. (2013). Correction of the distortions in the EPI images was implemented using B0-field maps obtained from double-echo FLASH acquisitions (matrix size, 64 × 64; 64 slices; spatial resolution, 3 mm^3^; short TE = 10 ms; long TE = 12.46 ms; TR = 1020 ms) and processed using the FieldMap toolbox in SPM (Hutton et al., 2002).

Preprocessing of structural and fMRI data was conducted using SPM12 (Wellcome Centre for Human Neuroimaging, University College London). All images were first bias-corrected to compensate for image inhomogeneity associated with the 32-channel head coil (Van Leemput et al., 1999). Fieldmaps were collected and used to generate voxel displacement maps. EPIs for each session were then realigned to the first image and unwarped using the voxel displacement maps calculated above. The three high-resolution structural images were averaged to reduce noise and coregistered to the whole-brain structural FLASH scan. EPIs were also coregistered to the whole-brain structural scan and spatially smoothed using a Gaussian smoothing kernel of 4 × 4 × 4 mm full-width at half maximum.

#### Statistical analyses

##### Behavioral data.

Data from eye tracking, in-scan vividness ratings, postscan memory tests, and debrief ratings were analyzed using repeated-measures ANOVAs (SPSS 17.0, SPSS) with a significance threshold of *p* < 0.05. Where Mauchly's test indicated that the assumption of sphericity had been violated, degrees of freedom were corrected using Greenhouse–Geisser estimates of sphericity.

##### fMRI data.

For data analysis, we used nonrotated task partial least squares (PLS), which is a multivariate method for extracting distributed signal changes related to varying task demands (McIntosh and Lobaugh, 2004; Krishnan et al., 2011). Data for each condition were included in a block design analysis and we conducted separate analyses for each of our contrasts of interest. Significance for each contrast was independently determined using a permutation test with 1000 permutations. We considered latent variables *p* < 0.05 as significant. The reliability of voxel saliences was assessed by means of a bootstrap estimation of the SE (McIntosh and Lobaugh, 2004). Bootstrapping is a sampling technique in which subjects are randomly selected into the analysis with replacement from the entire group of subjects. For each new sample, the entire PLS analysis is recalculated. In the present study, this sampling and analysis procedure was performed 500 times, resulting in estimates of the SE of the salience at each voxel. No corrections for multiple comparisons are necessary because the voxel saliences are calculated in a single mathematical step on the whole volume. We considered clusters of ≥10 voxels in which the bootstrap ratio was >1.96 (approximately equivalent to *p* < 0.05) to represent reliable voxels. In the current analyses, we specified a 14.6 s temporal window for each event (i.e., 4 TRs) to include the active phase of mental construction. Importantly, for each significant contrast reported in the main text, confidence intervals did not cross the 0 line, suggesting that each condition contributed to the pattern.

We used a large region of interest that included the whole medial temporal lobe (MTL)—hippocampus, entorhinal cortex (ENT), PRC, as well as PHC, RSC, and PCC, which have been implicated in scene processing. The mask also extended posteriorly to encompass regions of the early visual cortices (EVCs), including the precuneus (only inferior portions due to the partial volume), the calcarine sulcus, the lingual gyrus, and portions of the posterior fusiform cortex, given these regions have previously been implicated in different elements of mental imagery (Klein et al., 2000; Lambert et al., 2002; de Gelder et al., 2015).

## Results

Our main focus was on contrasts involving the Construct Scene task (Table 1). The contrast of primary interest was the Construct Scene task with the closely matched Construct Array task. As described above, these two conditions were well matched, requiring mental construction and associative processing of objects and space. The only difference between them was that the Construct Scene task required objects to be imagined on a 3D grid that gave rise to a scene-like representation (Fig. 1, compare *E*, *F*). Directly contrasting these tasks, therefore, allowed us to investigate brain regions that underpin scene construction while controlling for content, mental constructive, and associative processes.

**Table 1.** Results of the fMRI task comparisons

Task	Contrast	Brain regions	Hemisphere	MNI peak coordinate			Bootstrap ratio	Number of voxels
				x	y	z		
Construct Scene	> Construct Array (*p* = 0.004)	Anterior medial hippocampus–pre/parasubiculum	Right	22.5	−24	−16.5	3.7655	56
		Anterior calcarine sulcus (encompassing anterior lingual gyrus/PCC/RSC/inferior precuneus)	Left	−10.5	−51	6	3.3724	81
		Anterior calcarine sulcus (encompassing anterior lingual gyrus/PCC/RSC/inferior precuneus)	Right	9.0	−55.5	12.0	3.2213	102
		Posterior PHC	Right	28.5	−43.5	−10.5	2.8698	36
		Anterior medial hippocampus—pre/parasubiculum	Left	−21.0	−21.0	−19.5	2.8138	45
		Posterior fusiform cortex	Right	28.5	−33.0	−18.0	2.4450	20
	> Imagine Objects (*p* = 0.015)	Anterior lingual gyrus (encompassing inferior precuneus/PCC/RSC and extending posteriorly to encompass posterior lingual gyrus/posterior calcarine sulcus)	Right and left	10.5	−55.5	7.5	4.8465	3041
		Posterior PHC	Left	−30.0	−42.0	−10.5	4.0339	249
		Posterior hippocampus	Right	18.0	−37.5	1.5	3.9468	55
		Posterior PHC (extending anteriorly to encompassing anterior medial hippocampus— pre/parasubiculum)	Right	28.5	−42.0	−12.0	3.7299	360
		Anterior PHC (PRC)	Left	−33.0	−24.0	−34.5	3.6459	29
		RSC/PCC	Left	−7.5	−39.0	6.0	3.0623	16
		Anterior medial hippocampus—pre/parasubiculum	Left	−25.5	−24.0	−16.5	2.9320	52
		Posterior fusiform cortex	Right	21.0	−60.0	−12.0	2.2978	14
	> Imagine 3D Grid (*p* = 0.005)	Anterior lingual gyrus (encompassing anterior calcarine sulcus/inferior precuneus/PCC/RSC)	Right	9	−54	9	6.0388	341
		Inferior precuneus (encompassing PCC/RSC/anterior calcarine sulcus/anterior lingual gyrus)	Left	−4.5	−55.5	10.5	5.7412	314
		Posterior PHC	Left	−24	−37.5	−19.5	5.5618	723
		Posterior PHC	Right	25.5	−34.5	−21	4.6426	488
		Anterior PHC (PRC)	Left	−31.5	−6	−36	3.6334	89
		Anterior medial hippocampus—uncus	Left	−12	−9	−25.5	3.3703	10
		Anterior PHC (PRC)	Right	30	−3	−40.5	3.3075	110
		Anterior medial hippocampus—uncus	Right	22.5	−7.5	−25.5	2.6372	26
Construct Array	> Construct Scene (*p* = 0.004)	Anterior lingual gyrus (extending anteriorly to encompass posterior hippocampus and extending posteriorly to encompass posterior lingual gyrus/posterior calcarine sulcus)	Left and right	−16.5	−51.0	−4.5	4.9728	3479
		Anterior PHC (PRC/ENT)	Left	−28.5	−1.5	−39.0	3.8068	181
		Anterior PHC (PRC/ENT)	Right	28.5	3.0	−43.5	3.6093	440
		Anterior PHC (ENT/abutting the anterior medial hippocampus)	Left	−19.5	−12.0	−27.0	3.1719	119
		Anterior PHC (ENT)	Left	−19.5	4.5	−36.0	2.7904	27
		Anterior lingual gyrus	Right	4.5	−63.0	4.5	2.5501	10
	> Imagine Objects (*p* = 0.005)	Posterior calcarine sulcus (encompassing posterior lingual gyrus extending anteriorly to encompass anterior lingual gyrus/posterior PHC/posterior hippocampus)	Right and Left	10.5	−81.0	4.5	6.4355	6992
		Anterior PHC (PRC)	Right	24.0	1.5	−39.0	3.6467	47
		Posterior PHC	Right	34.5	−36.0	−13.5	2.4383	10
		Anterior PHC (ENT)	Left	−21.0	−16.5	−30.0	2.3777	16
		Anterior PHC (PRC)	Left	−28.5	−4.5	−37.5	2.3342	17
		Middle lateral hippocampus—CA1	Left	−34.5	−25.5	−16.5	2.3212	15
	> Imagine 2D Grid (*p* < 0.001)	Posterior PHC (extending anteriorly to encompass ENT/PRC)	Left	−30	−27	−24	5.3247	1014
		Posterior PHC (extending anteriorly to encompass ENT/PRC)	Right	31.5	−24	−22.5	4.5307	522
		Inferior precuneus (encompassing anterior calcarine sulcus/PCC/RSC/posterior hippocampus)	Left	−6	−52.5	12	4.1098	488
		Posterior hippocampus (encompassing inferior precuneus/PCC/RSC)	Right	15	−39	3	4.0605	279
		Anterior PHC (PRC)	Right	22.5	0	−49.5	3.3109	73
		Anterior lingual gyrus	Left	−24	−54	−3	2.7134	21
Imagine Objects	> Construct Scene (*p* = 0.015)	Anterior PHC (ENT/PRC)	Right	21.0	−9.0	−33.0	3.4047	241
		Anterior lateral hippocampus—CA1	Right	33.0	−10.5	−27.0	3.3312	11
		Anterior PHC (PRC)	Left	−25.5	−3.0	−39.0	3.2742	34
		Anterior PHC (ENT/abutting the anterior medial hippocampus)	Left	−18.0	−10.5	−27.0	3.2310	126
	> Construct Array (*p* = 0.005)	Anterior lateral hippocampus–CA1	Right	30.0	−13.5	−21.0	3.0863	42
Imagine 3D grid	> Construct Scene (*p* = 0.005)	Posterior lingual gyrus (encompassing posterior calcarine sulcus extending anteriorly to anterior lingual gyrus)	Left and right	−10.5	−78	−10.5	5.2507	3088
		Posterior fusiform cortex	Right	27	−55.5	−18	3.4263	33
		Anterior calcarine sulcus	Left	−22.5	−66	9	3.3149	24
		Anterior lingual gyrus	Right	27	−52.5	−6	3.1481	134
		Anterior lingual gyrus	Left	−22.5	−45	−1.5	3.102	14
		Anterior lingual gyrus	Left	−33	−54	−6	3.0345	13
		Anterior PHC (ENT)	Right	15	−6	−31.5	2.8463	73
		Anterior PHC (ENT)	Left	−18	−4.5	−31.5	2.4347	27
	> Imagine 2D Grid (*p* = 0.191)	No significant between-task differences	-	-	-	-	-	-
Imagine 2D grid	> Construct Array (*p* < 0.001)	Posterior lingual gyrus (encompassing posterior calcarine sulcus extending anteriorly to anterior lingual gyrus/anterior calcarine sulcus)	Left and right	−16.5	−72	−10.5	5.38	2134
		Anterior calcarine sulcus	Right	22.5	−61.5	10.5	3.9023	45
		Posterior calcarine sulcus		0	−90	−4.5	3.4177	20
		Anterior hippocampus	Right	28.5	−19.5	−15	2.6862	36
		Anterior lingual gyrus	Right	24	−46.5	−10.5	2.6018	27
		Anterior calcarine sulcus	Left	−21	−66	6	2.3526	22
	> Imagine 3D grid(*p* = 0.191)	No significant between-task differences	-	-	-	-	-	-

Bootstrap ratio (reliability of voxel saliences): >1.96 = *p* < 0.05; >2.58 = *p* < 0.01; >3.29 = *p* < 0.001.

Note that *p* values in the “Contrast” column relate to condition differences following permutation testing of the latent variables.

### fMRI task comparisons

**Figure 2.**
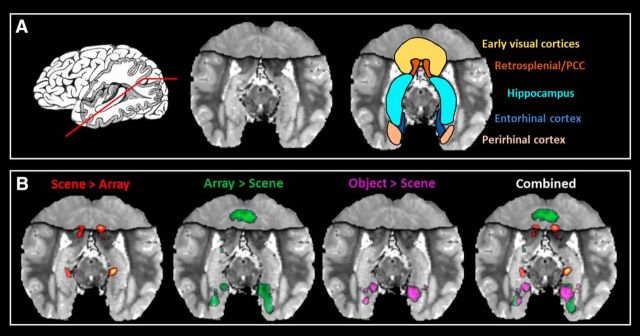
Results of the fMRI analysis. ***A***, Left, Representation of the oblique angle cutting through the hippocampus that we use to present the results in the other panels. Middle, Averaged structural MR image of the participants exposing the length of the hippocampus. Right, Regions of particular interest. ***B***, Left to Right, Results for the contrast of Construct Scene > Construct Array, Construct Array > Construct Scene, Imagine Object > Construct Scene, and all of the results combined. Results are thresholded at *p* < 0.05.

**Figure 3.**
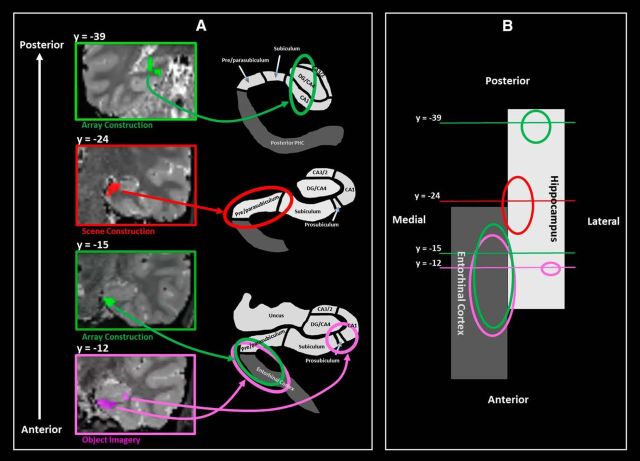
Summary of the main hippocampal activations. ***A***, The location of the left posterior hippocampal activation for the Construct Array > Construct Scene contrast (left, top). The right anterior medial hippocampal activation—pre/parasubiculum—observed for the contrast of Construct Scene > Construct Array (left, second panel from top). The entorhinal region abutting the much more anterior pre/parasubiculum recruited for both Construct Array (left, third panel from top) and Imagine Objects (left, bottom panel) tasks more so than Construct Scenes. The right anterior lateral hippocampal activation—prosubiculum/CA1—for the contrast of Imagine Objects > Construct Scene (left, bottom). The right panels show representative schematics of the locations of the hippocampal subregions. ***B***, Schematic representation of the hippocampus (white) and entorhinal cortex (gray) in the axial plane. The location of each of the coronal plane images presented in ***A*** is shown along with representations of the extent of each cluster.

Comparison of the Construct Scene task with the Construct Array task revealed that, in line with our prediction, a circumscribed region of the anterior medial hippocampus (peak voxel at *y* = −24) encompassing the pre/parasubiculum was preferentially recruited, bilaterally, during scene construction along with the PHC, RSC, and PCC (Figs. 2*B*, 3). Interestingly, the opposite contrast showed that array construction, more than scene construction, engaged the bilateral ENT, PRC, and EVC, and the left posterior hippocampus, which was part of a larger cluster of activity that encompassed the anterior lingual gyrus and portions of the EVC. This contrast also revealed activation of the left ENT abutting the anterior medial hippocampus (peak voxel at *y* = −12), which was more anterior to the pre/parasubiculum engaged by scene construction (Figs. 2*B*, 3).

The contrast of Construct Scene with Imagine Objects provided further support that the bilateral pre/parasubiculum along with the PHC, RSC, and PCC were specifically associated with scene construction (Fig. 3; Table 1). The reverse contrast showed that the mental construction of objects, more so than scenes, was associated with bilateral PRC and ENT. The right anterior lateral hippocampus, encompassing prosubiculum/CA1, and a left ENT activation that abutted the anterior medial hippocampus were also engaged. This area was more anterior (peak voxel at *y* = −10.5) to that associated with scene construction (Figs. 2*B*, 3).

The contrast of Construct Scenes with the Imagine 3D Grid revealed increased engagement of an anterior medial portion of the hippocampus in the approximate location of the uncus (peak voxel at *y* = −9) and the bilateral PRC. The reverse contrast showed that the mental construction of 3D grids, more so than scenes, was associated with the bilateral ENT and the posterior portions of the EVC. Imagine 3D Grid did not evoke increased hippocampal activation compared with Imagine 2D Grid, suggesting that 3D space alone was insufficient to engage the hippocampus.

To summarize (Fig. 3), our experimental design allowed us to parse the hippocampus and related areas dependent on the process that was being engaged. Circumscribed portions of the bilateral pre/parasubiculum (*y* = ∼−24) were specifically recruited during scene construction. By contrast, a more anterior portion of the ENT that abutted the anterior medial hippocampus was engaged during both array and object construction. Of note, these activated regions were clearly distinct (explicit smoothing, 4 mm; Euclidean distance between peak voxels of the Construct Scene vs Construct Array contrasts, 11.89 mm; Construct Array vs Imagine Object contrasts, 13.64 mm). The construction of mental images of arrays was also associated with increased activity in the posterior hippocampus as part of a larger cluster, which encompassed the lingual gyrus and the EVC. Object construction engaged the anterior lateral hippocampus in the region of prosubiculum/CA1. Outside the hippocampus, the PHC, RSC, and PCC were preferentially recruited for scene construction, compared with array construction. In contrast, compared with scene construction, array construction was associated with more posterior portions of the EVC, while both array and object construction were more strongly associated with the ENT and PRC.

But could other factors have influenced the results? We conducted a range of further analyses to investigate.

### Did participants truly engage with the tasks?

The construction of mental imagery cannot be directly measured. We therefore used a combination of methods to assess task attentiveness and compliance. First, we included catch trials where participants had to press a button if, during any trial, they heard a repeated phrase. On average, 94% (SD, 0.09) of catch trials were correctly identified, indicating that participants remained attentive throughout the experiment.

**Figure 4.**
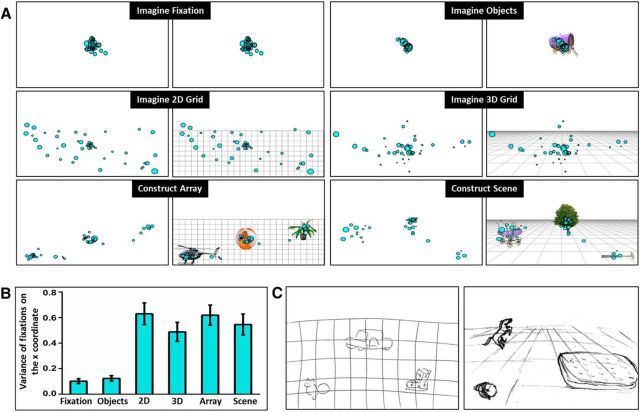
Eye movements and examples of postscan drawings. ***A***, Representative examples of fixation locations (cyan circles) during the imagination phase of a single trial of each task are overlaid on a blank white screen on which participants focused their imagination (left). A visual representation of what participants were asked to imagine on the screen is shown to the right of each panel. Note the central focus of fixations for the Imagine Fixation and Imagine Objects tasks, the more dispersed pattern over the bottom two-thirds of the screen for the Imagine 2D Grid and Imagine 3D Grid tasks and the three clusters for both the Construct Array and Construct Scene tasks representing the locations of the imagined objects. ***B***, The mean variance (±1 SEM) of fixations on the *x* coordinate during the imagination phase of each task. ***C***, Representative examples of postscan drawings for the Construct Array (left) and Construct Scene (right) tasks.

Second, we used in-scan eye tracking to ensure participants were performing each task according to the instructions. Visualization of fixations confirmed that participants engaged in each task according to our instructions (Fig. 4*A*). To formally determine the extent of eye movements, we measured the variance of all fixations in the horizontal axis during the construction phase of each trial (Fig. 4*B*). We predicted that if participants performed the mental-imagery tasks as expected, there would be less variance in fixation location during the center-focused Imagine Fixation and Imagine Objects tasks and a more dispersed pattern of fixations across the other tasks. The results of a repeated-measures ANOVA revealed a significant difference in the variance of fixations between tasks (*F*_(2.28,47.77)_ = 26.22, *p* < 0.001). In line with our prediction, *post hoc* analyses revealed significantly less variance during the Imagine Fixation task compared with the other tasks (compared with the Imagine 2D Grid, *t*_(21)_ = 6.286, *p* < 0.001; Imagine 3D Grid, *t*_(21)_ = 5.296, *p* < 0.001; Construct Array, *t*_(21)_ = 6.247, *p* = 0.001; Construct Scene, *t*_(21)_ = 5.839, *p* < 0.001). Significantly less variance was also observed in the Imagine Objects task compared with the other tasks (Imagine 2D Grid, *t*_(21)_ = 6.241, *p* < 0.001; Imagine 3D Grid, *t*_(21)_ = 4.949, *p* < 0.001; Construct Array, *t*_(21)_ = 6.266, *p* < 0.001; Construct Scene, *t*_(21)_ = 5.336, *p* < 0.001). There was no difference between the Imagine Fixation and Imagine Objects tasks (*t*_(21)_ = 1.702, *p* = 0.806). Variance during the Imagine 2D Grid task was significantly less than during the Imagine 3D Grid task (*t*_(21)_ = 3.819, *p* = 0.015). No other significant between-task differences were observed, including between the Construct Scene and Construct Array tasks (*t*_(21)_ = 1.897, *p* = 0.884).

Together, these measures provide quantitative evidence that participants paid attention during the experiment and engaged in mental-imagery construction in accordance with task instructions.

After scanning, we also asked participants to draw how they had imagined the arrays and scenes during the fMRI tasks. Examples are shown in Figure 4*C* and also indicate that participants complied with task requirements. The drawings also show that, despite both being composed of three objects related to a space, there was a clear representational difference between the arrays and the scenes. Further informative measures were obtained during postscan testing and debriefing, and these are described in following sections.

### Did other eye-movement features contribute to between-task differences?

To investigate the possibility that between-task differences in neural recruitment may be explained by other eye-movement features, we investigated the number of fixations, the duration of fixations, the number of saccades, saccade amplitudes, and scan paths, with a specific focus on our tasks of interest: Construct Array and Construct Scene. There were no differences in terms of the number of fixations (*t*_(21)_ = 0.144, *p* = 1.00), fixation durations (*t*_(21)_ = 0.423, *p* = 1.00), the number of saccades (*t*_(21)_ = 0.033, *p* = 1.00), or saccade amplitudes (*t*_(21)_ = 1.822, *p* = 0.726).

To examine scan paths, we split the screen into three equal areas of interest (AOIs)—left, middle, and right—and plotted the scan path for each trial. Two variables were measured to provide an index of the spatial distribution of scanning: the number of fixations and the dwell time within each AOI. Analyses showed that there was a task by AOI interaction for number of fixations (*F*_(1.249,26.237)_ = 5.989, *p* = 0.016), with Construct Array associated with more fixations to the right of the screen (*t*_(21)_ = 2.251, *p* = 0.035, *d* = 0.16), Construct Scenes associated with more fixations in the middle (*t*_(21)_ = 2.175, *p* = 0.041, *d* = 0.24), with no difference for the left side of the screen (*t*_(21)_ = 1.784, *p* = 0.089). Neither of these effects survived Bonferroni correction, and the effect sizes (Cohen's *d*) were small. There was also a task by AOI interaction for dwell time (*F*_(1.329,27.918)_ = 4.0, *p* = 0.045), with Construct Scene associated with a longer dwell time in the middle (*t*_(21)_ = 2.369, *p* = 0.027, *d* = 0.19), with no difference for the left of the screen (*t*_(21)_ = 0.772, *p* = 0.449) or the right of the screen (*t*_(21)_ = 1.840, *p* = 0.080). This effect did not survive Bonferroni correction, and the effect size was small.

Overall, therefore, the Construct Array and Construct Scene tasks were generally well matched, making it unlikely that between-task differences in neural recruitment were related to eye movements.

### Was hippocampal recruitment related to mnemonic processing?

Once out of the scanner after completing the experiment, participants were given two surprise memory tests (see Materials and Methods). Given the large number of stimuli and the fact that there was no explicit instruction to encode information—rather the emphasis was on mental construction, and memory was never mentioned—our expectation was that performance would be poor, even at chance, on the memory tests. We felt it was necessary, however, to conduct these tests in case successful encoding differed across tasks, and this could have explained differences in the brain areas that were engaged. Scores (means, SD) are shown in Table 2.

**Table 2.** Item and associative memory test performance (percentage correct)

	Task	Mean ± SD
Item memory	Imagine Object	82.85 ± 11.68
	Construct Array	76.03 ± 13.47
	Construct Scene	71.07 ± 11.87
	Identifying Novel Items	81.94 ± 11.17
Associative memory intact triplets	Imagine Object	51.09 ± 14.18
	Construct Array	55.16 ± 21.04
	Construct Scene	51.90 ± 13.38
Associative memory recombined triplets	Imagine Object	46.20 ± 17.45
	Construct Array	54.35 ± 19.44
	Construct Scene	48.91 ± 19.91

For the item memory test, participants performed above chance at recalling stimuli from the Imagine Objects (*t*_(22)_ = 13.491, *p* < 0.001), Construct Array (*t*_(22)_ = 9.268, *p* < 0.001) and Construct Scene (*t*_(22)_ = 8.514, *p* < 0.001) tasks, and were above chance at identifying novel items (*t*_(22)_ = 13.710, *p* < 0.001). The good performance on this test (with task means between 70 and 83% correct; Table 2) is a further indication that the participants paid attention, encoded information, and were engaged by the tasks. A repeated-measures ANOVA revealed a significant between-task effect on the item memory test (*F*_(1.77, 38.98)_ = 9.524, *p* < 0.001). *Post hoc* analyses showed that participants were better at recognizing object descriptions presented in the Imagine Objects task than objects presented in the Construct Array (*t*_(22)_ = 4.829, *p* < 0.001) and Construct Scene (*t*_(22)_ = 7.210, *p* < 0.001) tasks. Participants were also better at recognizing novel items than objects presented in the Construct Scene task (*t*_(22)_ = −3.382, *p* = 0.016). Notably, there was no significant difference between the Construct Array and Construct Scene tasks (*t*_(22)_ = 2.707, *p* = 0.075).

On the very challenging associative memory task, participants, as we expected, did not perform above chance on the recognition of intact triplets from the Imagine Objects (*t*_(22)_ = 0.368, *p* = 0.717), Construct Array (*t*_(22)_ = 1.177, *p* = 0.252), and Construct Scene (*t*_(22)_ = 0.682, *p* = 0.503) tasks. A repeated-measures ANOVA showed that there were no significant differences between the tasks for either the recognition of intact object triplets (*F*_(2,44)_ = 0.870, *p* = 0.426) or correct rejection of recombined object triplets (*F*_(2,44)_ = 1.651, *p* = 0.204). This included no significant difference between Construct Array and Construct Scene tasks (intact triplets: *t*_(22)_ = 1.047, *p* = 0.667; recombined triplets: *t*_(22)_ = 1.124, *p* = 0.616).

Overall, these results revealed no significant differences in memory performance in particular between our two tasks of interest: Construct Array and Construct Scene. Therefore, the differences we observed in neural recruitment during fMRI cannot be accounted for by differences in encoding success. Of note, it was not appropriate to run a subsequent memory analysis on the fMRI data using the individual object stimuli. This is because the three object descriptions that comprised one trial were presented in quick succession and it was not possible with fMRI to reliably tease apart signals relating to the specific items within a trial. Considering associative memory for the triplets, given that performance was not above chance in the subsequent surprise memory test, and that participants expressed low confidence about their memory decisions, using these data to interpret the fMRI data would be ill-advised. Moreover, in the associative memory test, two-thirds of the triplets were tested intact, but one-third of triplets were recombined to act as lures. Therefore, any subsequent memory fMRI analysis would likely be underpowered.

### Can imagery vividness account for hippocampal engagement?

During fMRI scanning, participants rated the vividness of mental imagery on each trial (see Materials and Methods; Fig. 1*H*; Table 3). Results of the repeated-measures ANOVA revealed a significant between-tasks difference in vividness ratings (*F*_(2.70,78.26)_ = 11.60, *p* < 0.001). *Post hoc* analyses showed that mental imagery during the Imagine Objects task was rated as more vivid than during the Imagine Fixation (*t*_(29)_ = 4.095, *p* = 0.005), Imagine 2D Grid (*t*_(29)_ = 5.586, *p* < 0.001), Imagine 3D Grid (*t*_(29)_ = 4.195, *p* = 0.004), Construct Array (*t*_(29)_ = 4.506, *p* < 0.001), and Construct Scene (*t*_(29)_ = 3.265, *p* = 0.041) tasks. Imagery during the Construct Array task was rated significantly more vivid than during the Imagine 2D Grid task (*t*_(29)_ = 3.398, *p* = 0.029). Imagery during the Construct Scene task was rated significantly more vivid than during the Imagine 2D Grid (*t*_(29)_ = 4.116, *p* = 0.004) and Imagine 3D Grid (*t*_(29)_ = 3.224, *p* = 0.046) tasks. Importantly, no significant difference was observed between the Construct Array and Construct Scene tasks (*t*_(29)_ 2.116, *p* = 0.483).

**Table 3.** Subjective measures

Question	Rating (Yes/No or 1–5 Likert scale)		Tasks of relevance	Mean ± SD
How vivid was your imagination of the xxxx? (From the trial-by-trial vividness ratings during scanning)	1, not vivid at all	5, extremely vivid	Imagine Fixation	3.77 ± 0.65
			Imagine Object	4.16 ± 0.53
			Imagine 2D grid	3.69 ± 0.62
			Imagine 3D grid	3.84 ± 0.57
			Construct Array	3.96 ± 0.52
			Construct Scene	4.03 ± 0.53
How difficult was it to imagine the xxxx?	1, very easy	5, very difficult	Imagine Fixation	2.58 ± 1.22
			Imagine Object	2.01 ± 0.75
			Imagine 2D grid	2.65 ± 1.09
			Imagine 3D grid	2.13 ± 0.88
			Construct Array	2.35 ± 0.76
			Construct Scene	2.43 ± 0.85
How often did you engage in off-task thoughts (mind-wander) during this task?	1, never	5, always	Imagine Fixation	2.22 ± 0.80
			Imagine Object	1.77 ± 0.57
			Imagine 2D grid	2.30 ± 0.99
			Imagine 3D grid	1.87 ± 0.67
			Construct Array	1.88 ± 0.65
			Construct Scene	1.95 ± 0.83
In general, how detailed do you think your images of the xxxx were?	1, hardly any details at all	5, extremely richly detailed	Imagine Object	3.27 ± 0.45
			Imagine 2D grid	2.97 ± 0.81
			Imagine 3D grid	3.22 ± 0.67
			Construct Array	3.27 ± 0.69
			Construct Scene	3.45 ± 0.62
In general, did you feel that you successfully imagined the objects in a scene?	1, never successful	5, always successful	Construct Scene	3.77 ± 0.84
Did you feel that you imagined the objects in a scene at all and had to try to repress this?	1, never had to repress	5, always repressed	Construct Array	1.28 ± 0.69
On a scale of 1 to 5, how 2D/3D was your mental imagery during this task?	1, completely 2D	5, completely 3D	Construct Array	1.90 ± 0.84
			Construct Scene	4.10 ± 0.82
Did you make up any stories or scenarios related to the xxxx?	0, no; 1, yes		Imagine Object	0.02 ± 0.09
			Imagine 2D grid	0.07 ± 0.25
			Imagine 3D grid	0.02 ± 0.09
			Construct Array	0.03 ± 0.18
			Construct Scene	0.07 ± 0.25
Did you keep your viewpoint fixed?	0, no; 1, yes		Imagine 2D grid	0.98 ± 0.09
			Imagine 3D grid	0.97 ± 0.18
			Construct Array	0.97 ± 0.18
			Construct Scene	0.98 ± 0.09
To what extent did you imagine other objects or details in addition to the given objects? Did you … ?	1, think of objects with other details the entire time	5, not think of other objects or details at all	Imagine Object	4.07 ± 0.83
			Construct Array	4.23 ± 0.73
			Construct Scene	4.08 ± 0.77

### Can perceived task difficulty or subjective differences in mental imagery account for hippocampal recruitment?

In the debriefing session after scanning and after completion of the surprise memory tests, participants were asked about their experience of performing each task (Table 3). Participants reported that they could perform the tasks with ease with no between-task differences for perceived task difficulty (*F*_(3.37,97.85)_ = 2.396, *p* = 0.066; including no difference between the Construct Array and Construct Scene tasks, *t*_(29)_ = 0.524, *p* = 1.00). Significant between-task differences were observed for the rating of mind-wandering (*F*_(3.46,100.39)_ = 3.638, *p* = 0.011). *Post hoc* analyses showed that compared with the Imagine Objects task, participants were more prone to engage in mind-wandering during the Imagine Fixation (*t*_(29)_ = 3.465, *p* = 0.025) task. This makes sense considering the fixation task was included as a rest condition for participants. There was no significant difference between Construct Array and Construct Scene tasks (*t*_(29)_ = 0.436, *p* = 1.00). Significant differences were also observed on the rating of detail of mental imagery (*F*_(3.47, 100.70)_ = 3.510, *p* = 0.014). *Post hoc* analyses showed that mental imagery during the Construct Scene task was significantly more detailed than during the Imagine 2D Grid task (*t*_(29)_ = 3.093, *p* = 0.043). No other significant between-task differences were observed, including between Construct Array and Construct Scene tasks (*t*_(29)_ = 1.884, *p* = 0.514).

Participants further confirmed (Table 3) that, during the Construct Scene task, they were successful at creating a scene in their mind's eye. In contrast, participants reported a clear sense of imagining objects on a 2D grid during the Construct Array task and stated that they rarely felt a need to repress mental imagery of scenes during this task. Direct comparison showed that, as expected, the Construct Scene task was rated as subjectively more 3D than the Construct Array task, which was rated as more 2D (*t*_(29)_ = 11.988, *p* < 0.001). There were no significant differences between the tasks (including between the Construct Array and Construct Scenes tasks) on several other subjective measures: the creation of narratives about the stimuli (*F*_(2.15,62.43)_ = 0.597, *p* = 0.565; Construct Array vs Construct Scene, *t*_(29)_ = 1.00, *p* = 0.981), the fixedness of the viewpoint (*F*_(1.96,56.75)_ = 0.139, *p* = 0.867; Construct Array vs Construct Scene, *t*_(29)_ = 0.441, *p* = 0.999), and the inclusion of extraneous objects or other details (*F*_(2,58)_ = 0.957, *p* = 0.390; Construct Array vs Construct Scene, *t*_(29)_ = 1.055, *p* = 0.657).

In summary, subjective measures indicated that the participants performed the task with ease and followed instructions. As might be expected, there were some minor differences. For example, increased mind-wandering was noted during the Imagine Fixation task. However, no significant differences were found between the Construct Array and Construct Scene tasks.

### Results summary

The results of the fMRI analyses revealed that when other associative and mental constructive processes were taken into account, a specific region of the anterior medial hippocampus—corresponding to the location of the pre/parasubiculum—was engaged during scene construction along with other regions that have previously been linked to scene processing, including the PHC, RSC, and PCC. In contrast, array construction was more strongly associated with the ENT, the PRC, and the posterior portions of the EVC, and with activation within the left posterior hippocampus and left ENT abutting the anterior medial hippocampus. Importantly, this latter activation was in a location more anterior to the cluster observed during scene construction. Interestingly, the Imagine Objects task resulted in activation of the anterior lateral hippocampus. The differing patterns of neural recruitment between the very tightly matched Construct Array and Construct Scene tasks could not be accounted for by differences in eye movements, mnemonic processing, or the phenomenology of mental imagery.

## Discussion

The aim of this study was to compare accounts that place associative processes at the heart of hippocampal function with the theory that proposes scene construction as one of its primary roles. Using closely matched tasks during high-resolution fMRI, we found that, as predicted, the pre/parasubiculum in the anterior medial hippocampus was preferentially engaged by the construction of scenes (three objects and a 3D space). However, it was also evident that different regions within the hippocampus were engaged by the construction of arrays (three objects and a 2D space) that did not evoke scene representations. In this case, the posterior hippocampus and an ENT region that abutted the anterior hippocampus were recruited. Even the imagination of object triplets that had no spatial context activated this latter region along with an anterior portion of the lateral hippocampus in the approximate location of prosubiculum/CA1. Overall, these results show that one possible reason for ongoing debates about how the hippocampus operates may be because it does not only process space or associations or scenes. Instead, there may be multiple processing circuits within the hippocampus that become engaged, depending on task demands.

Our primary contrast of interest was array construction compared with scene construction. These tasks were closely matched on stimulus content and mental constructive and associative processes. Attention, eye movements, encoding success, and perceived difficulty did not differ between them. Phenomenologically, the vividness and detail of their imagery were also matched. Nevertheless, in line with our prediction and with previous reports, a circumscribed portion of the pre/parasubiculum in the anterior medial hippocampus was preferentially involved in scene construction (Maass et al., 2014; Zeidman et al., 2015a,b; Hodgetts et al., 2016; Zeidman and Maguire, 2016). Importantly, our findings reveal for the first time that this region is preferentially recruited, not for mental construction per se, not for imagining a 3D space alone, but specifically for mental construction of scenes couched within a naturalistic 3D spatial framework.

Drawing on the latest anatomical evidence, Dalton and Maguire (2017) recently noted that the pre/parasubiculum is a primary target of the parietomedial temporal processing pathway and may receive integrated information from foveal and peripheral visual inputs (Kravitz et al., 2011). Thus, it has privileged access to holistic representations of the environment and so could be neuroanatomically determined to preferentially process scenes. Indeed, Dalton and Maguire (2017) suggest the pre/parasubiculum may be the hippocampal “hub” for scene-based cognition. The PHC, RSC, and PCC are also implicated in the anatomical scene-processing network connecting with the pre/parasubiculum. Aligning with this evidence and their known links with scene processing (Epstein, 2008), we found that these brain areas were more engaged during scene compared with array construction.

By contrast, array construction engaged a different set of brain areas, namely, the ENT, PRC, and posterior portions of EVC, with the left ENT/PRC cluster extending to abut the anterior medial hippocampus and the EVC cluster extending anteriorly along the lingual gyrus into the left posterior hippocampus. Where the activity involved the ENT and abutted the hippocampus, the location bordered the pre/parasubiculum much more anteriorly than that for scene construction. Therefore, naturalistic 3D scene representations may engage a circumscribed portion of the anterior pre/parasubiculum in unison with the PHC, RSC, and PCC. By contrast, more general or abstracted forms of spatial imagery, such as objects on a 2D space (Constantinescu et al., 2016), might recruit a more anterior portion of the ENT abutting the very anterior pre/parasubiculum. The different parts of the hippocampus and distinct cortical regions engaged by scenes and arrays, despite the close matching of the tasks, precludes the view that scenes are merely being enabled by processing sets of associations similar to those underpinning array construction. What we document here are separable mental construction processes giving rise to distinct types of representation in and around the hippocampus.

Considering the posterior hippocampal activation during array construction, this area has been implicated in a broad range of cognitive processes (Poppenk et al., 2013; Strange et al., 2014; Zeidman and Maguire, 2016), including spatial memory (Moser and Moser, 1998; Maguire et al., 2006) and mnemonic processing of items in a 2D space (de Rover et al., 2011). While our results reflect involvement of the posterior hippocampus in mental imagery of objects in a 2D rather than a 3D space, it is unlikely that the posterior hippocampus is only involved in this form of mental imagery. The anatomy of the most posterior portion of the human hippocampus is particularly complex (Dalton et al., 2017), with much still to learn. Ultrahigh-resolution MRI investigations at the level of subfields are required to further inform our understanding of posterior hippocampal contributions to specific cognitive processes.

The Construct Objects task was not designed to be a close match for the array and scene tasks, but was included to inform about the brain areas engaged during object construction and object-only associations, where spatial context was irrelevant. Of note, vividness of the imagery and memory for the objects in this task was significantly better than those in the array and scene construction tasks. Therefore, any fMRI results should be interpreted with this is mind. PRC recruitment during object imagery would be predicted, and indeed was found, considering the strong association between the PRC and object-centered cognition (Murray et al., 2007). Overall, the object task engaged very similar areas to those recruited for array construction, namely, the PRC and the ENT abutting the very anterior medial left hippocampus. This may reflect a generic area for nonscene-based associative processing. Where object construction differed from both array and scene tasks was in the activation of the right anterior lateral hippocampus in the region that aligns with the location of the prosubiculum/CA1. This finding is concordant with the prediction of Dalton and Maguire (2017), based on neuroanatomical considerations, where the PRC, ENT, and prosubiculum/CA1 are heavily interconnected (Insausti and Muñoz, 2001; Li et al., 2017). Therefore, as with the array and scene construction tasks, the mental construction of isolated objects engaged a differentiable portion of the hippocampus.

Our results show that for associations between objects, between objects and 2D space, or between objects and 3D space, the hippocampus does not seem to favor one type of representation over another; it is not a story of exclusivity. Rather, there may be different circuits within the hippocampus, each associated with different cortical inputs, that become engaged, depending on the nature of the stimuli and the task at hand. This may explain why it has been so difficult to reconcile hippocampal theories that have generally focused on one type of process or representation. Our results may also explain why disparate patterns of cognitive preservation and impairment are reported in patients with bilateral hippocampal lesions. For any individual, damage may affect particular subregions within the hippocampus more than others. These subtle case-by-case differences in the microscopic topography of damage and sparing may affect cognition in different ways that as yet remain undetectable by current MRI technology.

While some theoretical accounts have posited that distinct areas within the MTL may preferentially process specific types of representation (Barense et al., 2005; Graham et al., 2010), perhaps surprisingly, such representational differences have not typically been extended to processing within the hippocampus. Nonhuman primate tract tracing studies have shown clear differences in how different cortical and subcortical brain regions interact not only with specific hippocampal subfields (DeVito, 1980; Rockland and Van Hoesen, 1999; Ding et al., 2000), but also disproportionately with specific portions of subfields along the longitudinal (anterior–posterior) and transverse (distal–proximal) axes of the hippocampus (Insausti and Muñoz, 2001). In recent years, functional differentiation down the long axis of the hippocampus (Nadel et al., 2013; for review, see Poppenk et al., 2013; Strange et al., 2014; Zeidman and Maguire, 2016) and subfield-specific hypotheses (Libby et al., 2012; Berron et al., 2016; Guzman et al., 2016;) have received increasing attention. Our results further emphasize the importance of considering the hippocampus as a heterogeneous structure, and that a focus on characterizing how specific portions of the hippocampus interact with other brain regions may promote a better understanding of its role in cognition.

It remains possible that other factors may have affected our results. For example, it could be that participants engaged in more size transformation of objects, or visualization of objects in a more distant space, during the Construct Scene task. We are, however, unaware of any evidence for MTL involvement in these processes (Larsen et al., 2000). Future work is needed to precisely characterize the different information-processing streams within the human hippocampus, both anatomically and functionally. Presumably these circuits are linked, but how and to what extent will also be important questions to address. In humans, little is known about intrahippocampal functional connectivity or even connectivity between specific hippocampal subfields and the rest of the brain. Use of ultrahigh-resolution fMRI is clearly warranted to help move beyond an “either/or” view of the hippocampus to a more nuanced understanding of its multifaceted contributions to cognition.
